# Acquired reactive perforating collagenosis triggered by trauma with eosinophilia: a case report and literature review

**DOI:** 10.3389/fmed.2024.1415545

**Published:** 2024-06-26

**Authors:** Jie Liu, Peng Zhao, Xinzhong Zhang, Jie Gao, Haozhi Han, Junxia Qin

**Affiliations:** ^1^The Dermatology Department of Shanxi Provincial People’s Hospital, Five Hospital of Shanxi Medical University, Taiyuan, China; ^2^Department of Dermatology, Universitätsklinikum Erlangen, Friedrich-Alexander-Universität Erlangen-Nürnberg, Erlangen, Germany; ^3^Department of Minimally Invasive Spine Surgery, The Affiliated Shanxi Provincial People’s Hospital of Shanxi Medical University, Taiyuan, China

**Keywords:** ARPC, eosinophilia, skin rash, traumatic trigger, case report

## Abstract

Acquired reactive perforating collagenosis (ARPC) is a rare dermatological disorder condition defined by the perforation of altered collagen fibers through the epidermis. The presence of underlying conditions such as diabetes or renal disease is helpful in the ARPC diagnosis. Although skin rashes related to ARPC have been reported, the exact causative factors and mechanisms remain unclear. Here, we present a unique case of ARPC triggered by trauma in a 67-year-old male without concurrent systemic alterations. The diagnosis of ARPC with eosinophilia was made following comprehensive diagnostic testing, including clinical presentation, histological results, and blood tests, ruling out other possible diseases. Intriguingly, the histopathological examination revealed collagen penetration into the epidermis at different tissue sections. In addition, we reviewed existing literature on ARPC, which documented the causation. To help confirm the diagnosis, clinicians have to pay attention to traumatic triggers for ARPC and its rare manifestation with eosinophilia.

## Introduction

Reactive perforating collagenosis (RPC), first described by Mehregan et al. (1), is typically considered a rare dermatological condition disorder defined by the transepidermal elimination of altered collagen fibers through the epidermis. It can be classified into two variants: inherited RPC (IRPC) and acquired RPC (ARPC) (2). ARPC is more frequently observed in adult population and is commonly associated with underlying systemic conditions, notably diabetes mellitus, chronic kidney disease, and hypertension (3). It can also be associated by cirrhosis, pulmonary fibrosis, hypothyroidism or hyperthyroidism, and malignant tumors, with reports of ARPC occurring during pregnancy (4). Additionally, drugs such as efalizumab, infliximab, and rituximab, as well as skin irritation from mosquito bites, hemodialysis, and laser hair removal, can induce the onset of this disease, with hemodialysis being the most significant risk factor (5). However, cases of ARPC caused by trauma without any underlying disease and increased eosinophilia in patients have been rarely reported.

We present a case of ARPC with eosinophilia. The uniqueness of this case lies in the ARPC being attributed to a scratch from a branch, the absence of commonly associated systemic conditions, such as diabetes mellitus and chronic kidney disease, and the presence of peripheral eosinophilia. We believe that providing an updated review of ARPC in the medical literature would help clarify certain uncertain points regarding its etiology, differential diagnosis, and pathogenesis.

## Case description

**Figure 1:**
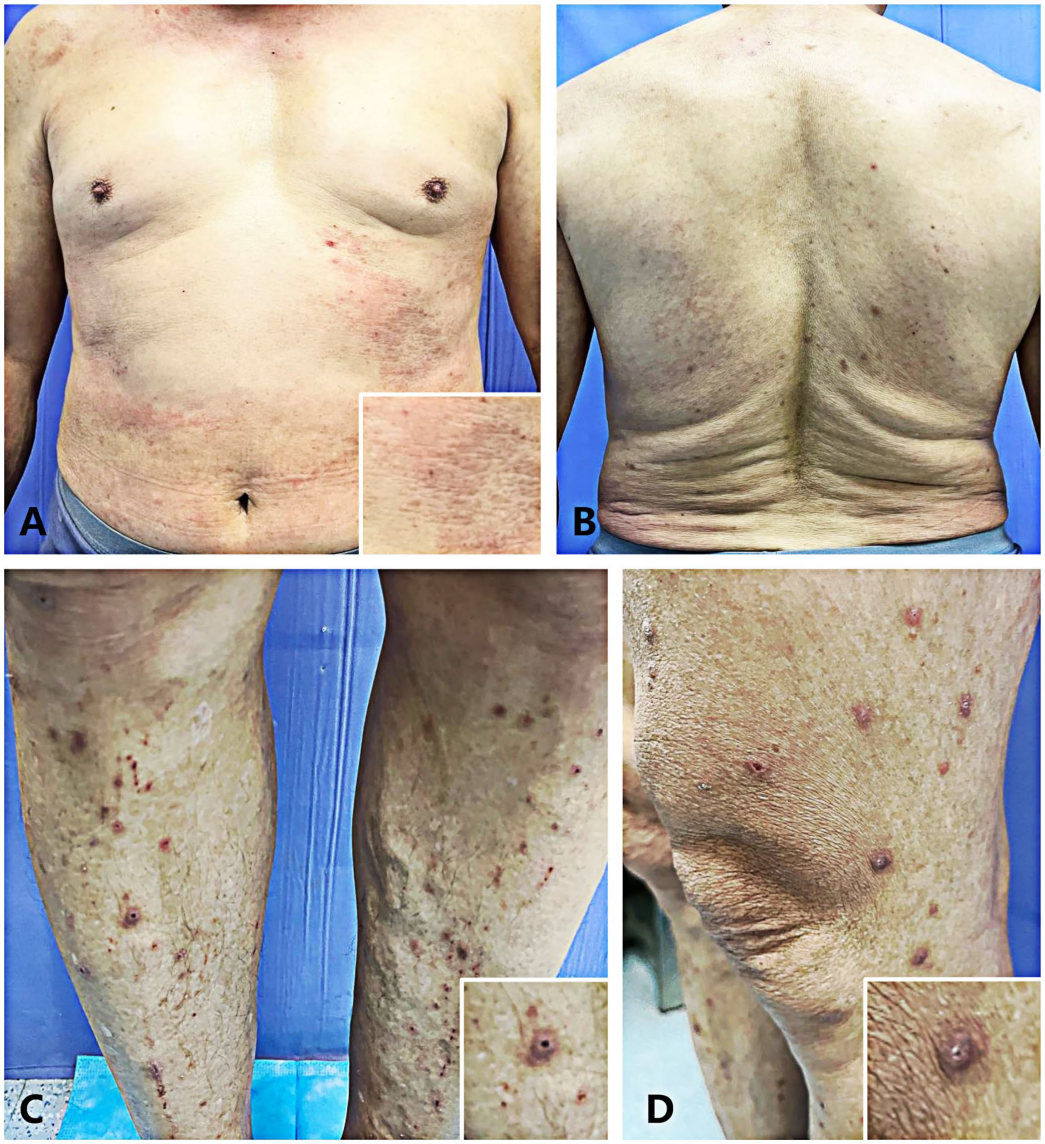
**(A,B)** The patient’s lesions presented as flat reddish macules and papules on the abdomen **(A)** and trunk **(B)**. **(C,D)** The patient presented with multiple well-circumscribed keratotic papules bilaterally distributed on the lower extremities.

A 67-year-old male patient presented to the dermatology outpatient clinic with a chief complaint of intensely pruritic papules lesions on his bilateral lower limbs, which had persisted for 2 months. The patient reported subsequent spread of the lesions to his trunk and abdomen (Figure 1). He recalled that the onset was subsequent to a trauma from a branch while exercising without using medicine or having comorbidities before the onset of this episode, resulting in several erythematous, itchy cutaneous lesions at the same location. Following that, scattered papules and nodules appeared, causing severe itching and sleep disturbances. There was no reported history of diabetes mellitus, hypertension, or chronic renal disease in this family (although genetic analysis was not obtained). He sought treatment at an external clinic multiple times and received topical medication (specifics unknown), with no significant improvement observed. On physical examination, crateriform lesions with an erythematous base and crusty substance inside (Figures 1C,D), along with similar but smaller lesions on his trunk and abdomen (Figures 1A,B). Routine serum laboratory tests revealed in all four tests the absolute eosinophil count > 0.63 × 10^9^/L (normal reference range 0.05 × 10^9^/L to 0.5 × 10^9^/L) and a percentage of blood eosinophils > 7.7% (normal reference range 0.5 to 5%). Other routine serum laboratory investigations, including liver function tests, renal function tests, serum glucose level, urinalysis and stool exam, were all within normal limits.

**Figure 2:**
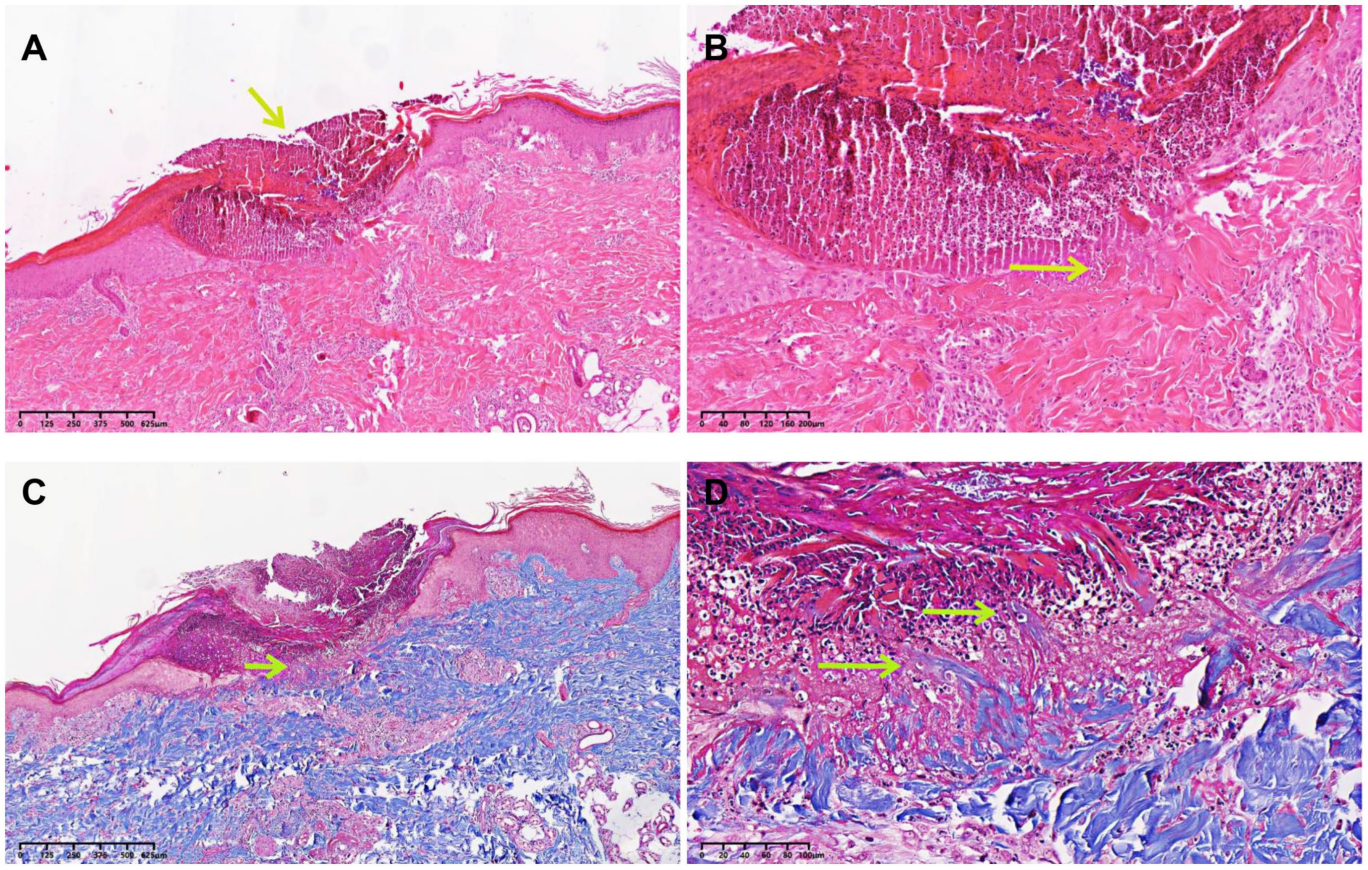
Histopathological examination of the biopsy specimen. **(A)** A skin biopsy sample from the papule located on the lower extremity demonstrated degenerated collagen bundles that had been eliminated to form a cup-shaped epidermal depression (HE, ×10, arrow). **(B)** High-power view highlighted the degenerating collagen bundles, vertically oriented, and the basophilic debris within the crater. Numerous neutrophils and lymphocytic infiltration were seen directly under the depression (HE, ×100, arrow). **(C)** Collagen bundles perforated the base of the epidermal erosion (Masson’s trichrome staining, ×10, arrow). **(D)** High-power view highlighted the degenerating collagen bundles, vertically oriented, and the basophilic debris within the crater (Masson’s trichrome staining, ×100, arrows).

A skin biopsy was performed on a representative lesion located on the lower extremity. Histopathological examination revealed degenerated collagen bundles that had been eliminated to form a cup-shaped epidermal depression (Figure 2A). High-power view highlighted the degenerating collagen bundles, vertically oriented, and the basophilic debris within the crater. Numerous neutrophils and lymphocytic infiltration were seen directly under the depression (Figure 2B). The findings were consistent with reactive perforating collagenosis, as evidenced by the presence of collagen elimination before (Supplementary Figures 1A,B) and after complete penetration through the epidermis (Figures 2A,B; Supplementary Figures 1C,D). Masson’s trichrome staining showed that collagen bundles perforate the base of the epidermal erosion (Figure 2C). High-power view highlighted the degenerating collagen bundles, vertically oriented, and the basophilic debris within the crater (Figure 2D).

A diagnosis of ARPC with eosinophilia was established. Treatment with topical halomethasone monohydrate at a dosage of 3 g per day for 2 weeks, in combination with oral antihistamine therapy consisting of levocetirizine at 5 mg per day and tripterygium glycoside at 60 mg per day for a duration of 5 weeks. This therapeutic regimen effectively alleviated the patient’s pruritus and prevented further scratching of the affected areas. After 2 months of treatment, the skin lesions showed significant resolution, with only temporary post-inflammatory hyperpigmentation remaining at the original lesion sites. The patient was informed of the importance of regular follow-up and continued to be monitored accordingly.

## Discussion

ARPC caused by trauma without any underlying disease and increased eosinophilia in patients have been rarely reported. Mehregan et al. (1) suggested that trauma in genetically susceptible individuals leads to collagen necrobiosis in the dermal papillae. Herzinger et al. (6) showed that immune activation may be involved in the development of ARPC, with collagen types IV and VII from the basal membrane identified in the plug of ARPC skin lesions. Other studies suggested that the transepidermal elimination of collagen may be a response to chronic scratching or rubbing in pruritic conditions (7). The investigation of ARPC is of paramount importance for several reasons, including its rare documentation in the published literature, the crucial role of histopathological examination in establishing the diagnosis, and its frequent association with underlying systemic disorders (8). Consistent with the literature (9), our patient presented with a disseminated cutaneous eruption involving the abdomen, trunk, and lower extremities. Histopathological examination of the skin biopsy revealed a well-circumscribed area of necrosis containing a keratotic plug. Within this necrotic region, parakeratotic cells and lymphocytic infiltration were observed. Notably, sparse collagen fibers in the dermis were found to perforate the epidermis, forming a cup-shaped structure surrounded by acanthosis and hyperkeratosis of the epidermis. Furthermore, a lymphocytic infiltrate was present in the superficial dermis and perivascular regions. Intriguingly, the histopathological examination captured the penetration of collagen into the epidermis at different tissue sections.

The differential diagnosis for this case primarily includes Kyrle’s disease (KD), perforating folliculitis (PF), and elastosis perforans serpiginosa (EPS) (7, 10), while ARPC, KD, PF, and EPS share the common features of transepidermal elimination of altered dermal components, the differentiation is made based on the types of epidermal damage and the characteristics of the eliminated material. Distinguishing ARPC from other perforating dermatoses can be challenging, and the clinical presentation and histopathological examination are crucial for accurate diagnosis. In KD, the lesions typically present as papules filled with conical keratotic plugs, often located near hair follicles. The pathological characteristics of KD include epidermal invagination, visible eosinophilic material fragments, absence of elastic fiber tissue, and a perforating base with granulomatous inflammatory reaction (11, 12). ARPC lesions are typically larger and deeper, while KD or PF manifests as smaller and more numerous rashes. The biggest difference between KD and ARPC is the presence of a granulomatous inflammatory reaction in KD, which is absent in ARPC (13). PF presents with erythematous, follicular papules measuring a few millimeters across, with a white, central keratotic plug. The lesions are disseminated and primarily found on hair-bearing extremity surfaces (14). The pathological manifestations of PF include significantly dilated hair follicles, filled with incompletely keratinized keratotic plugs, fragments of eosinophilic material, and coiled hair. In the present case, the skin lesions did not exhibit perifollicular growth, and histopathological examination revealed the absence of discernible follicular structures. ARPC and EPS are distinct skin conditions with different characteristics. ARPC is characterized by large, deep lesions, often associated with systemic diseases such as diabetes or chronic renal failure. In contrast, EPS presents with small papular lesions that form a serpiginous pattern and is frequently associated with connective tissue disorders like pseudoxanthoma elasticum. Additionally, ARPC typically affects adults, while EPS is more common in children and young adults (15). In addition, Alves et al. reported a case of perforating granuloma annulare (perforating GA) that should be considered in the differential diagnosis of ARPC (16). The pathological manifestations of perforating GA show progressively degenerated dermal collagen fibers, surrounded by a large number of epithelioid cells and infiltrated by other chronic inflammatory cells, whereas ARPC show no evidence of the epithelioid cells. Their case did not support a perforating collagenosis, which generally presents as multiple keratotic papules on the extensor surfaces of the limbs (17).

We reviewed cases of ARPC reported in the literature and summarized their causes, clinical characteristics, complications, and prognoses (Table 1). The typical clinical presentation of RPC involves multiple round plaques and nodules with central hyperkeratotic plugs, most commonly distributed on the extremities and back, with occasional involvement of the face and neck. Pruritus is a frequent symptom associated with RPC. Interestingly, in contrast to previous reports, the Koebner phenomenon was not mentioned in the majority of cases reviewed in this study (29). Among the reported patients with RPC, the most frequently associated comorbidities included diabetes mellitus, chronic kidney failure (with or without dialysis), lymphoma, IgA nephropathy, and acquired immunodeficiency syndrome (AIDS). However, it is noteworthy that the exact etiological factors were not specified in the majority of the reported cases (18, 30–32). Kim and Ghosh reported cases of ARPC developing following insect bites (5, 33). Miyazaki et al. described a case of ARPC and eosinophilic granulomatosis with polyangiitis (EGPA) occurring concurrently in a patient with diabetes (28). Their findings suggest that the increased secretion of matrix metalloproteinases (MMPs) in patients with EGPA may contribute to collagen degeneration and deposition in the context of diabetes. Furthermore, they hypothesized that this process could be associated with the transepidermal elimination of altered collagen, potentially facilitated by the weakening of the basement membrane. In contrast to the case reported by Miyazaki et al., our patient presented with ARPC induced by trauma from a branch, accompanied by eosinophilia. Notably, the patient had no family history of similar dermatological conditions or other systemic disorders, such as diabetes mellitus and renal failure, which have been previously associated with RPC. Therefore, this is the first reported case of ARPC induced by trauma combined with eosinophilia without other systemic disorders. Consequently, establishing a diagnosis of ARPC based solely on the presence of other systemic diseases poses a challenge in this case.

**Table 1:** Reported cases of ARPC with a clear causative factor and accompanying symptoms.

Patient No.	Publication year	Age (yrs)/Gender	Lesion duration	Distribution of skin lesions	Causes	Complications	Treatment	Outcomes	Ref.
1	1998	25-year-old male	1 month	Face, trunk, and both forearms	Alteration of the capillaries in the dermis	IgA nephropathy.	Corticosteroid ointment and oral administration of an antihistamine and antibiotic.	Not effective	Iwamoto et al. (18)
2	2007	29-year-old male	6-weeks	Lower back and buttocks	Insect bite	No systemic association	Three cycles of intralesional triamcinolone acetonide injections administered biweekly, combined with daily topical application of desoxymethasone 0.25% ointment.	Following 3 months of treatment, the lesions resolved completely, with temporary post-inflammatory hyperpigmentation observed at the sites of the initial lesions.	Kim et al. (5)
3	2009	22-year-old female	4 months	Arms, forearms, and hands	Insect bite	No systemic association	Oral antihistamines and topical tretinoin (0.05%) twice daily.	Within 6 weeks the lesions started to resolve.	Ghosh et al. (5)
4	2012	55-year-old female	4 months	Lower extremities	Subcutaneous insulin injections	Type 2 diabetes mellitus	Management of her diabetes mellitus and underwent narrow UVB treatment.	Without significant amelioration.	Ataseven et al. (19)
5	2013	74-year-old female	Unknown	Head and neck, trunk and extremities	Scratching	Dermatomyositis; HCV	Unknown	Unknown	Kikuchi et al. (20)
6	2013	56-year-old female	Unknown	The lesions were located on the eyelids, the lateral aspects of the second fingers, and the face.	Scratching	Dermatomyositis	Unknown	Unknown	Kikuchi et al. (20)
7	2016	41-year-old male	2 weeks	Forehead and forearms	Induced by extensive sun exposure	Hemodialysis patient	The patient was initially treated with topical steroids and subsequently received combination therapy consisting of allopurinol and N-acetylcysteine (NAC).	After 2 months of treatment, most lesions had resolved (see Supporting Information).	Sabanis et al. (21)
8	2020	68-year-old male	Unknown	The lesions were distributed on the chest, arms, back, neck, and buttocks.	The lesions were suspected to be secondary to a drug reaction, erythema multiforme, or bullous pemphigoid.	Stage IV CKD	The patient was initially treated with intravenous solumedrol 60 mg, hydroxyzine, and a combination of doxycycline 100 mg and dapsone. On day 7, the solumedrol was switched to prednisone to initiate a tapering regimen.	The patient’s pain was effectively managed throughout the treatment course. Upon receiving the biopsy results, doxycycline and dapsone were discontinued. Subsequently, the lesions began to form scabs, which ultimately sloughed off, leaving residual scars.	Kochen et al. (22)
9	2021	23-year-old male	3 months	Lower limbs, upper limbs	Tinea pedis infection	Fungal infection	Itraconazole 200 mg BID for 8 weeks was administered.	Following the resolution of the lesions, no recurrence of the ulcers was observed. However, multiple scars formed at the sites of the previous lesions.	Ye et al. (23)
10	2021	62-year-old female	Unknown the exact time	Back and extremities	Scratching panitumumab	Advanced colon cancer	The cessation of panitumumab and irinotecan.	Two months after treatment, the pruritic eruption had resolved, leaving residual scarring and mild pigmentation at the affected sites.	Tsutsui et al. (24)
11	2021	71-year-old male	1 year	Trunk and limbs	Scratching	Atopic dermatitis	Monotherapy of dupilumab in a routine way.	After 3 months of treatment, the patient experienced significant improvement in both skin lesions and pruritus. Notably, no adverse effects were reported by the patient throughout the treatment period.	Ying et al. (25)
12	2021	70-year-old male	2 years	Trunk and limbs	Scratching	Atopic dermatitis	The patient was initiated on dupilumab monotherapy, with an initial dosage of 600 mg, followed by a maintenance dose of 300 mg administered twice monthly.	Eruptions and itching improved substantially.	Ying et al. (25)
13	2022	31-year-old female	2 weeks	The lesions were distributed on the lower extremities, upper extremities, scalp, face, and buttocks.	Induced by hydroxychloroquine	The patient had a history of undifferentiated connective tissue disease, characterized by fatigue and arthralgia.	Systemic antihistamines (diphenhydramine 50 mg orally, twice daily) and topical steroid cream (betamethasone dipropionate cream). Additionally, oral isotretinoin 20 mg daily was initiated. The patient was also advised to undergo phototherapy as part of the treatment plan.	There were no new skin lesions or systemic features.	Alenzi et al. (26)
14	2022	75-year-old female	5 years	The lesions were located on the trunk, right upper extremity, and left lower extremity.	MRSA	Diabetes	The patient was treated with topical corticosteroids and oral antihistamines to manage the skin lesions and associated pruritus. Additionally, medications were administered to achieve. Optimal glucose control, given the patient’s underlying diabetes.	Following treatment, the keratin plug decreased in size, and the patient experienced relief from pruritus.	Huang et al. (27)
15	2024	70-year-old male	Unknown	Trunk	Scratching	Diabetes; Eosinophilic granulomatosis with polyangiitis developing simultaneously	Combination therapy of prednisolone, minocycline and diaphenylsulfone was successful.	Successful treatment.	Miyazaki et al. (28)

This case presents several interesting aspects. Firstly, the patient did not have a significant family history of similar lesions, which initially was not given much consideration. Secondly, the patient reported a history of trauma from scratching by a tree branch preceding the onset of the rash. We hypothesize that ARPC could have been triggered by increased eosinophils due to exposure to microorganisms or insect eggs on the tree branch, although concrete evidence to support this theory is lacking. Notably, previous literature has documented cases where insect bites led to the development of ARPC (5, 33). The pathogenesis of ARPC remains incompletely understood. Mehregan et al. (1) initially proposed that superficial trauma may contribute to the development of ARPC. Ghosh et al. (5) reported a case of ARPC following an insect bite and considered the transepidermal elimination of collagen as a reaction pattern resulting from chronic scratching or rubbing in a subset of pruritic patients. Additionally, Miyazaki et al. (28) showed a case of ARPC and eosinophilic granulomatosis with polyangiitis (EGPA) developing concurrently in a diabetic patient, suggesting that increased secretion of MMP-2 and MMP-9 in EGPA might be associated with collagen degeneration in the skin. Furthermore, although the pathogenesis of ARPC is unknown, overexpression of transforming growth factor-3 (TGF-β3) has been seen in many ARPC patients. TGF-β, matrix metalloproteinase-1, and tissue inhibitor of metalloproteinase-1 immunoreactivity were significantly increased in the lesions of ARPC (34), indicating the crucial function of these factors in regulating epidermal homeostasis, postponing the re-epithelialization and remodeling, and changing extracellular matrix protein metabolism (7). In the present case, mild superficial trauma or inflammatory cell infiltration may have contributed to the development of ARPC, resulting in the intense affinity for hematoxylin in the connective tissue of the papillary dermis or the wall of the superficial capillaries. Subsequently, parakeratosis and epidermal atrophy with transepidermal elimination of collagen occurred. Moreover, increased eosinophils may have been caused by exposure to microorganisms or insect eggs on the tree branch, potentially stimulating the secretion of MMPs (such as MMP-2 and MMP-9). The precise mechanisms underpinning the pathogenesis of ARPC require further investigation. Thirdly, common comorbid disorders typically associated with ARPC, such as hypothyroidism, hyperparathyroidism, dermatomyositis, or liver dysfunction, were absent in this case. However, the patient did not have a long-standing history of diabetes or renal failure. Fourth, it is worth mentioning that the absolute eosinophil count in all four tests was >0.63 × 10^9^/L (normal reference range 0.05 × 10^9^/L to 0.5 × 10^9^/L) and the percentage of blood eosinophils was >7.7% (normal reference range 0.5% to 5%). Reports suggest that eosinophils, along with neutrophils, can release MMPs upon stimulation by tumor necrosis factor-α (TNF-α) (35). This heightened MMP secretion could potentially contribute to collagen degeneration in the skin in this specific case.

## Conclusion

We report a unique case of ARPC triggered by trauma and accompanied by eosinophilia, in the absence of concurrent systemic disorders. To the best of our knowledge, this represents the first documented case of ARPC associated with eosinophilia and lacking a familial history or systemic conditions such as diabetes mellitus and renal failure. Further investigations are necessary to validate the potential link between ARPC and trauma-induced eosinophilia. Additionally, elucidating the underlying mechanism and causal relationship between eosinophilia and ARPC warrants comprehensive exploration.

## Data availability statement

The original contributions presented in the study are included in the article/Supplementary material, further inquiries can be directed to the corresponding author.

## Ethics statement

Written informed consent was obtained from the individual(s) for the publication of any potentially identifiable images or data included in this article. Written informed consent was obtained from the participant/patient(s) for the publication of this case report.

## Author contributions

JL: Validation, Writing – original draft. PZ: Data curation, Investigation, Supervision, Writing – review & editing. XZ: Methodology, Software, Writing – review & editing. JG: Investigation, Resources, Writing – review & editing. HH: Software, Writing – review & editing. JQ: Investigation, Supervision, Validation, Writing – review & editing.
