# Supplementary figures and images for: Acquired reactive perforating collagenosis triggered by trauma with eosinophilia: a case report and literature review
Source: Front Med (Lausanne). 2024 Jun 26;11:1415545. doi: 10.3389/fmed.2024.1415545 (PMC11233541; doi:10.3389/fmed.2024.1415545)

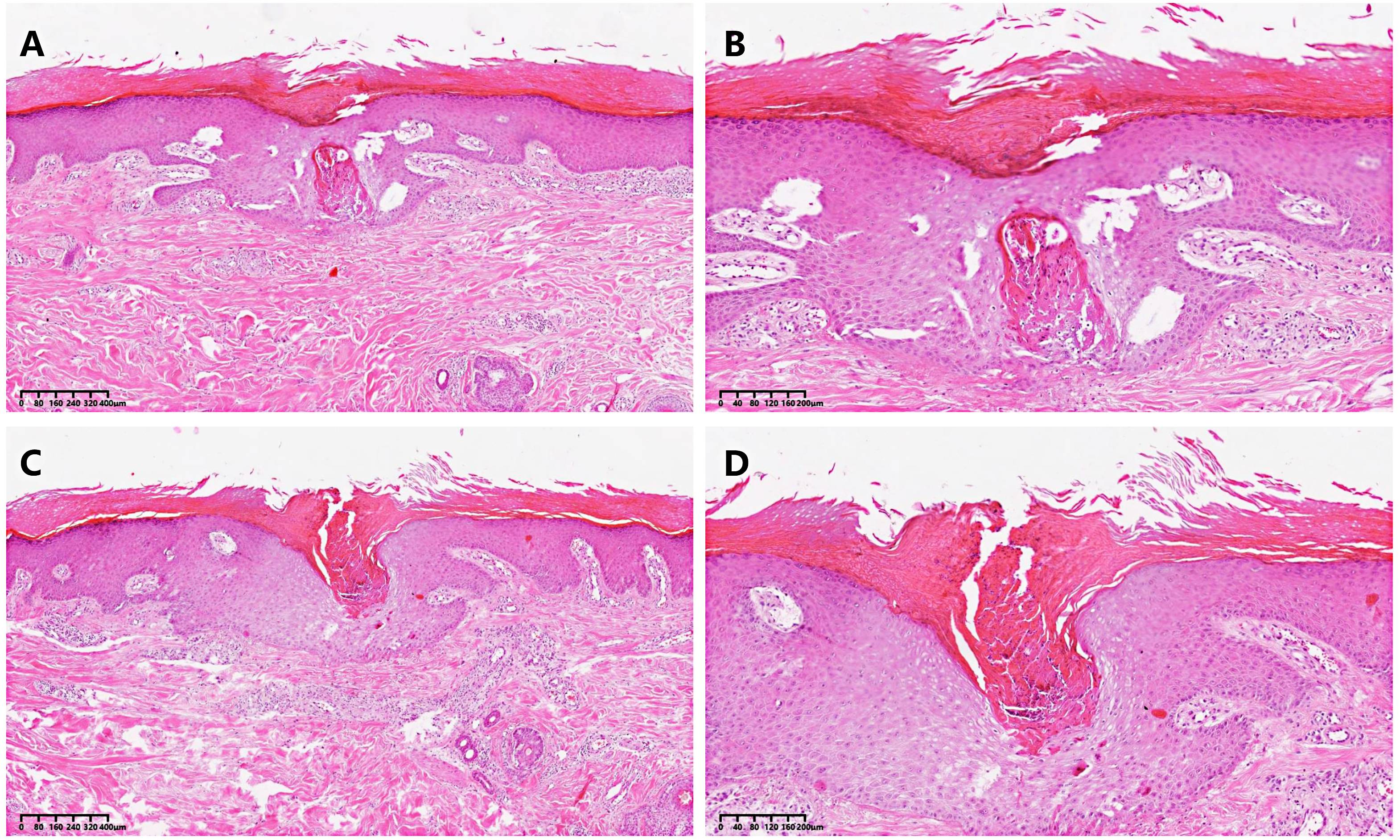

Supplement: Supplementary file 1 [file Image_1.JPEG]
